# Supplementary material for: Netcombin: An algorithm for constructing optimal phylogenetic network from rooted triplets
Source: PLoS One. 2020 Sep 18;15(9):e0227842. doi: 10.1371/journal.pone.0227842 (PMC7500971; doi:10.1371/journal.pone.0227842)
Supplement: S4 File — (ZIP) [file pone.0227842.s004.zip › SequenceData-Triplet/sequenceData-10-20-30-0-Triplet/length 400, numseq 30, rate 10^-7/1 seq.pdf]

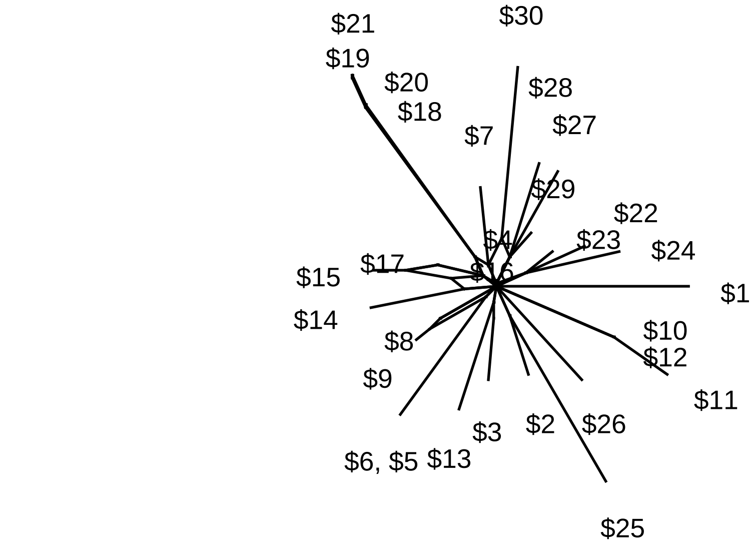
0.0010
$21
$30
$19
$20
$28
$18
$27
$7
$29
$22
$4
$23
$24
$17
$16
$15
$1
$14
$10
$8
$12
$9
$11
$3
$2
$26
$6, $5
$13
$25
